# Lifestyle and Subsequent Malignant Neoplasms in Childhood Cancer Survivors: A Report from the St. Jude Lifetime Cohort Study

**DOI:** 10.3390/cancers16050864

**Published:** 2024-02-21

**Authors:** Aron Onerup, Sedigheh Mirzaei, Shalini Bhatia, Maria Åberg, Megan E. Ware, Lenat Joffe, Lucie M. Turcotte, Chelsea G. Goodenough, Yadav Sapkota, Stephanie B. Dixon, Matthew D. Wogksch, Matthew J. Ehrhardt, Gregory T. Armstrong, Melissa M. Hudson, Kirsten K. Ness

**Affiliations:** 1 Department of Epidemiology and Cancer Control, St. Jude Children’s Research Hospital, Memphis, TN 38105, USAyadav.sapkota@stjude.org (Y.S.); melissa.hudson@stjude.org (M.M.H.); kiri.ness@stjude.org (K.K.N.); 2 Department of Pediatrics, Institute of Clinical Sciences, Sahlgrenska Academy, University of Gothenburg, 405 30 Gothenburg, Sweden; 3 Department of Biostatistics, St. Jude Children’s Research Hospital, Memphis, TN 38105, USA; 4 School of Public Health and Community Medicine, Institute of Medicine, University of Gothenburg, 405 30 Gothenburg, Sweden; maria.aberg@gu.se; 5 Region Västra Götaland, Regionhälsan, 413 45 Gothenburg, Sweden; 6 Department of Pediatrics, Zucker School of Medicine at Hofstra/Northwell, Hempstead, NY 11549, USA; 7 Department of Pediatrics, University of Minnesota, Minneapolis, MN 55455, USA; 8 Department of Oncology, St. Jude Children’s Research Hospital, Memphis, TN 38105, USA

**Keywords:** physical activity, fitness, body mass index, cancer, subsequent malignant neoplasms, childhood cancer, epidemiology

## Abstract

**Simple Summary:**

It has been shown that lifestyle factors such as smoking, alcohol consumption, diet, and physical activity affect the risk of developing cancer in older adults. While this is not the case for childhood cancers, survivors of childhood cancer are at increased risk of developing cancer in adulthood, called subsequent malignant neoplasms, due to the cancer treatment they received in childhood. We aimed to assess whether the risk of developing subsequent malignant neoplasms in young adulthood was associated with lifestyle factors. We could not see any association between lifestyle factors and subsequent malignant neoplasms in young adult childhood cancer survivors. This suggests that while lifestyle has other health benefits, it is possible that the risk of subsequent malignant neoplasms in young adult childhood cancer survivors cannot be modified with lifestyle behaviors.

**Abstract:**

Introduction: This study aimed to assess longitudinal associations between lifestyle and subsequent malignant neoplasms (SMNs) in young adult childhood cancer survivors. Methods: Members of the St. Jude Lifetime Cohort (SJLIFE) aged ≥18 years and surviving ≥5 years after childhood cancer diagnosis were queried and evaluated for physical activity, cardiorespiratory fitness (CRF), muscle strength, body mass index (BMI), smoking, risky drinking, and a combined lifestyle score. Time to first SMN, excluding nonmalignant neoplasms and nonmelanoma skin cancer, was the outcome of longitudinal analysis. Results: Survivors (*n* = 4072, 47% female, 29% smokers, 37% risky drinkers, 34% obese, and 48% physically inactive) had a mean (SD) time between baseline evaluation and follow-up of 7.0 (3.3) years, an age of 8.7 (5.7) years at diagnosis, and an age of 30 (8.4) years at baseline lifestyle assessment. Neither individual lifestyle factors nor a healthy lifestyle score (RR 0.8, 0.4–1.3, *p* = 0.36) were associated with the risk of developing an SMN. Conclusions: We did not identify any association between lifestyle factors and the risk of SMN in young adult childhood cancer survivors.

## 1. Introduction

With treatment success increasing rapidly in recent decades, there is a growing population of childhood cancer survivors (CCSs) at a relatively young adult age [1]. CCSs are at an increased risk of developing treatment-related subsequent malignant neoplasms (SMNs) [2]. Since CCSs cannot modify their treatment exposures after cure, identifying possible avenues of intervention to reduce the risk of SMN is important. There are reports of a higher prevalence of physical inactivity, overweight/obesity, and poor fitness in CCSs than in the general population [3,4].

Several lifestyle habits are associated with the risk of many adult cancers in the general population. Initially, tobacco smoking and alcohol consumption were identified [5,6], with evidence for associations between body mass index (BMI) [7,8,9], physical activity [10], and fitness [11] and adult cancer emerging over the past two decades. While there are indications that the associations between higher BMI and any cancer start already in the 30s [8], the current evidence for associations between BMI, physical activity/fitness, and cancer in young adulthood is weak. 

This study aimed to assess longitudinal associations between lifestyle-associated variables (physical activity, fitness, BMI, smoking, and risk drinking) and SMNs in childhood cancer survivors participating in the St. Jude Lifetime Cohort Study (SJLIFE) [12]. 

## 2. Materials and Methods

### 2.1. Population

The SJLIFE details and assessments have been previously described [12,13]. Participants gave written informed consent before participating in the study. The St. Jude Lifetime Cohort Study is conducted in accordance with the declaration of Helsinki and was approved by the Institutional Review Board at St. Jude Children’s Research Hospital, with a current approval date of 13 March 2023. For this analysis, participants were ≥5 years after diagnosis of childhood cancer. We used information on exposures from the first SJLIFE campus assessment at ≥18 years. 

### 2.2. Self-Reported Exposures

Physical activity was self-reported, as (MVPA min/day) and calculated into the metabolic equivalent task (MET) hours per week. Self-reported alcohol consumption was categorized as risky drinking for >3 drinks per day or >7 per week for women and >4 per day or >14 per week for men. Self-reported tobacco smoking was categorized as current, past, or never smoker. 

### 2.3. Objectively Assessed Exposures

CRF was assessed with the six-minute walk test, analyzing meters walked divided into quartiles [14]. Muscular strength was evaluated by knee extension strength (Nm/kg) and handgrip strength testing (kg). Knee extension strength was measured as peak torque from five repetitions at 60 degrees per second. The maximum value was used for analyzing both knee extension strength and handgrip strength. 

Height in centimeters and weight in kilograms were measured with a wall-mounted stadiometer (SECA, Hanover, MD, USA) and an electronic scale (Scale-tronix, White Plains, NY, USA), respectively. Body mass index was calculated (kg/m^2^) and categorized as underweight (BMI < 18.5), normal weight (BMI 18.5–24.9), overweight (BMI 25–29.9), or obesity (BMI ≥ 30). Fat and lean mass were measured with dual X-ray absorptiometry (QDR 4500, software version 13.3:3; Hologic, Bedford, MA, USA) in the total-body scanning mode. Percent body fat and percent lean mass were calculated by dividing fat mass and fat-free mass by total body mass. For men, 25–29% body fat was categorized as pre-obesity and ≥30% as obesity. For women, 30–34% was categorized as pre-obesity and ≥35% as obesity [15].

Participants were categorized according to a recently published lifestyle score [16]. Self-reported physical activity, BMI category, risky drinking (>3 drinks per day or >7 per week for women, >4 per day or >14 per week for men), and smoking status were given 0–1 point each and combined to create a lifestyle score (0–4) that was categorized as unhealthy (0–2), moderately healthy (2.5–3), or healthy (3.5–4). 

### 2.4. Childhood Cancer Treatment

Childhood cancer treatment exposures are ascertained in the SJLIFE from medical records, as previously described [12]. We included information on anthracyclines (doxorubicin equivalent dose) [17], alkylating agents (cyclophosphamide equivalent dose), platinum agents (cisplatin equivalent dose), epipodophyllotoxins, and radiation therapy.

### 2.5. Outcomes

As part of the comprehensive clinical evaluation in SJLIFE, a detailed medical history is obtained from all participants [12]. When neoplasms are reported, they are verified pathologically and/or radiologically, through medical records, or from National Death Index reports [18]. A physician categorizes all subsequent neoplasms as benign or malignant, in accordance with the fourth position of the morphology code in the International Classification of Diseases for Oncology (ICD-O), version 3 [19]. For the main analyses, we used any SMN (excluding nonmalignant neoplasms and nonmelanoma skin cancer) diagnosed at least one year after the baseline campus visit to reduce the risk for inverse causality, i.e., a low fitness due to current undiagnosed cancer. Since the association can be expected to differ by type of cancer, we also intended to analyze by type of SMN for the most common types of SMN (breast cancer, gastrointestinal cancer, and thyroid cancer). 

## 3. Statistical Analysis

All analyses were performed according to a prespecified statistical analysis plan, with covariates selected by a directed acyclic graph. Time to first SMN was the outcome of the analysis. Piecewise exponential models were used to evaluate the associations of lifestyle factors with time to the first SMN; the follow-up time started one year after the first assessment to reduce the risk of reverse causality. The results were given as rate ratios (RRs) with 95% confidence intervals (CIs). The analyses were adjusted for demographic (sex, age at diagnosis and baseline assessment) and treatment (anthracyclines, alkylating agents, epipodophyllotoxins, platinum agents, and radiation therapy) or diagnostic factors (childhood cancer diagnosis). Analyses were performed for the risk of developing any SMN and for subsequent breast cancers since there were too few events for the other types of SMN that are associated with lifestyle in the general population. 

## 4. Results

### 4.1. Baseline Characteristics

The study population included 4072 survivors followed for 7.0 (3.3) years after initial assessment at age 30 (8.4). Participant characteristics are shown in Table 1. Defined by BMI, 1384 (34%) were categorized as obese, while this was the case for 1602 (49%) when using assessments from the DEXA (Table 1). Participants reported a median of 9 MET h/week and the combined lifestyle score was categorized as unhealthy for 1941 (51%) participants. 

### 4.2. Incidence and Risk Factors for SMNs

Any SMN was experienced by 178 (4.4%) survivors, including 38 breast cancers, 28 gastrointestinal cancers, 17 urological cancers, and 41 thyroid cancers. For a more detailed list of the first SMN types experienced by each individual, see Supplemental Table S1. Comparison to population-based data from the Surveillance Epidemiology and End Results program indicates a standardized incidence ratio of 30.7 (95% CI 26.3–35.5). In the multivariate analyses, male sex (RR 0.6, 95% CI 0.5–0.8) and primary cancer diagnosis after 1990 (RR 0.4, 95% CI 0.2–0.8) were associated with lower SMN risk, while survivors of Hodgkin lymphoma (RR 2.5, 95% CI 1.4–4.5) or neuroblastoma (RR 3.1, 95% CI 1.3–7.5) and those treated with radiotherapy (RR 2.2, 95% CI 1.5–3.2) had a higher risk of SMNs compared to other survivors. For breast SMNs, the strongest risk factor was chest radiation (RR 3.33, 95% CI 1.66–10.0). In total, 186 participants died during follow-up, which was accounted for by censoring in the analyses, Supplementary Table S2.

### 4.3. Associations between Lifestyle and SMNs

In the univariate analyses, BMI-defined overweight (RR 1.5, 95% CI 1.0–2.2) was associated with a higher risk of developing any SMN (Table 2). However, this association was not seen in the adjusted analyses. Except for CRF and BMI, no association was seen between lifestyle variables and the risk of developing an SMN in the univariate or multivariate analyses (Table 2). For CRF, no association was seen in the univariate analyses. However, the multivariable analyses including treatment showed a higher risk of SMNs for participants in the third quartile of CRF compared to those in the first quartile (RR 1.7, 95% CI 1.0–2.6, Table 2). This association was not seen in the model adjusted for childhood cancer diagnosis instead of treatment (RR 1.3, 95% 0.8–2.1). Since 1/3 of the SMNs occurred in survivors of Hodgkin lymphoma, we explored the distribution of CRF in survivors of Hodgkin lymphoma compared to all other survivors. This showed that survivors of Hodgkin lymphoma had a higher CRF at baseline (Supplementary Table S3). Since the analyses included any SMN, radiation exposure was not specific to the SMN site, possibly resulting in confounding by Hodgkin lymphoma in the models adjusted for treatment. No associations were seen between the lifestyle factors and the risk of developing subsequent breast cancer (Table 2). No analyses could be performed for the other subtypes of SMN due to too few events. 

## 5. Discussion

In this observational study of 4072 childhood cancer survivors, we did not observe any association between lifestyle and SMNs. To our knowledge, there are no previously published longitudinal studies of the association between lifestyle factors and SMNs in childhood cancer survivors. 

The only previously published study on lifestyle and SMNs in childhood cancer survivors was a case–control study that reported a potential association between obesity during childhood cancer treatment and a higher risk of developing SMNs [20]. We could not find any significant association between BMI or any of the other lifestyle variables and SMNs in our analyses within the SJLIFE. There are several possible explanations for this difference. One is the timing of SMN onset, where the case–control study assessed early-onset SMNs, occurring a mean of 7 years after a primary cancer diagnosis, while our mean follow-up was 28 years after diagnosis. This is likely because cancers developed early after therapy are different than those developed years after therapy. Our most common SMN types were thyroid and breast cancer, while their most common was acute myeloid leukemia. Leukemia was one of the cancer types where we saw an association with BMI already at 20 years follow-up in our previous Swedish study [9], while this was not the case for several of the solid tumors, including thyroid cancer. Another possible explanation is that overweight/obesity during childhood has a stronger association with cancer, which has been suggested for the general population [8,21]. This should be further studied in childhood cancer survivors. 

Our results are surprising given the literature documenting associations between lifestyle factors and cancer in the general population [6,7,10]. There are some possible explanations for these discrepant findings. It is probable that the higher-than-expected SMNs experienced during follow-up in this relatively young population, indicated by a standardized incidence ratio of 30.7, is highly dependent on exposure to carcinogenic treatments during childhood and underlying genetic predisposition to cancer. Lifestyle may not be able to overcome the influence of treatment exposure on increased risk for SMNs in this population. Given the distribution of SMN types in this young population, it is also possible that while lifestyle does not appear to influence specific types of cancer treatment related SMN risk, lifestyle factors may influence cancer types not typically attributed to treatment exposures. As malignancies generally develop later in life in the general population than they do in younger adults of the age range in our study, the association between lifestyle and SMNs may not yet be detectable, likely varying with attained age in childhood cancer survivors. This hypothesis is supported by a recent report on lifestyle and cause-specific late mortality from the Childhood Cancer Survivor Study, with an increased risk for SMN mortality evident 35 years after diagnosis in those with a poor lifestyle [16]. This is also supported by our results from a Swedish population-based sample of men, showing no protective associations between CRF and any of the site-specific cancers diagnosed until age 38, corresponding to the age distribution in our SJLIFE population [11]. However, our other Swedish study also showed that BMI was associated with several malignancies diagnosed in patients’ 30s [9]. The only lifestyle measure that was associated with SMNs in the univariate analyses in our SJLIFE analysis was BMI, while the adjusted analyses did not show any significant association. 

This study has several strengths. The analyses were performed in the SJLIFE, with its detailed clinically ascertained assessments of both treatment exposures, functional assessments, possible confounders, and SMNs. The longitudinal follow-up and start of follow-up one year after the first SJLIFE assessment reduced the risk of reverse causality with poor lifestyle-associated variables due to cancer- or treatment-related impairments from the SMN. While the sample size was not as large as populations in the general population, a 4072 participant cohort is relatively large from a childhood cancer survivor perspective. The main limitation of this study was the low number of events due to a relatively short follow-up. This made it impossible to assess associations between lifestyle and specific types of SMN. A cross-sectional analysis including any SMN occurring before the first SJLIFE assessment would have increased power but would also have introduced a risk of reverse causality, with participants having undergone a second cancer treatment being at higher risk of poor performance and body composition. The rate ratios (RR) for lifestyle and any SMN was close to 1 for most lifestyle-associated variables and the models detected other expected risk factors for developing SMNs. However, it is possible that, e.g., the absence of an association between BMI and SMNs was a result of low statistical power. Longer follow-up of this cohort in whom lifestyle factors and adverse events including SMNs are ascertained over time will provide us with the opportunity to evaluate this association as survivors age. Similar analyses are also ongoing in the larger Childhood Cancer Survivor Study, with the potential of strengthening the evidence base on associations between physical activity, BMI, and SMNs.

## 6. Conclusions

We did not identify any association between lifestyle factors and the risk of SMNs in young adult childhood cancer survivors. While we are unable to conclude the reasons for our null results, possible explanations are both that the increased risk from treatment exposures for SMNs in young adult survivors cannot be modified by lifestyle and that lifestyle is associated more strongly with cancer diagnosed later in life.

## Figures and Tables

**Table 1:** Demographic, lifestyle, diagnosis, and treatment variables according to body composition.

	Developed Any SMN	No SMN	Total	P between Groups	Developed Breast Cancer	Developed Thyroid Cancer	Developed Gastrointestinal Cancer
Number of participants	178	3894	4072		38	41	28
Sex, female, *n* (%)	105 (59%)	1820 (47%)	1925 (47%)	0.001	36 (95%)	22 (54%)	18 (64%)
Age at baseline, years, mean (sd)	36.3 (8.9)	29.8 (8.3)	30.1 (8.5)	<0.001	38.3 (7.0)	34.4 (6.6)	36.0 (8.9)
Years of follow-up, mean (sd)	5.3 (2.9)	7.1 (3.3)	7.0 (3.3)	<0.001	6.4 (3.4)	4.8 (2.6)	5.8 (3.1)
Race/ethnicity, *n* (%)				0.075			
Non-hispanic white	156 (88%)	3101 (80%)	3257 (80%)		33 (87%)	38 (93%)	25 (89%)
Non-hispanic black	18 (10%)	605 (16%)	623 (15%)		3 (7.9%)	2 (4.9%)	2 (7.1%)
Hispanic	3 (1.7%)	111 (2.9%)	114 (2.8%)		2 (5.3%)	1 (2.4%)	1 (3.6%)
Other	1 (0.6%)	77 (2.0%)	78 (1.9%)		0	0	0
Educational attainment, *n* (%)				0.046			
<High school, high school graduate or ged	43 (25%)	1150 (32%)	1193 (31%)		7 (18%)	6 (15%)	10 (36%)
Training after high school or some college	60 (35%)	1316 (36%)	1376 (36%)		12 (32%)	17 (44%)	8 (29%)
College graduate or postgraduate	70 (40%)	1164 (32%)	1234 (32%)		19 (50%)	16 (41%)	10 (36%)
Missing	5	264	269		0	2	0
Household income per year, *n* (%)				0.31			
<USD 20,000	56 (37%)	1336 (44%)	1392 (43%)		9 (27%)	14 (42%)	12 (48%)
USD 20,000–USD 59,999	54 (36%)	999 (33%)	1053 (33%)		13 (39%)	11 (33%)	6 (24%)
≥USD 60,000	40 (27%)	723 (24%)	763 (24%)		11 (33%)	8 (24%)	7 (28%)
Missing	28	836	864		5	8	3
Body mass index, mean (sd)	28.9 (7.4)	28.2 (7.2)	28.2 (7.2)	0.10	28.8 (8.1)	29.1 (7.0)	28.6 (7.6)
Underweight (<18.5 kg/m^2^), *n* (%)	7 (3.9%)	143 (3.7%)	150 (3.7%)		3 (7.9%)	2 (4.9%)	0
Normal weight (18.5/24.9 kg/m^2^), *n* (%)	50 (28%)	1355 (35%)	1405 (34%)		10 (26%)	9 (22%)	12 (43%)
Overweight (25/29.9 kg/m^2^), *n* (%)	63 (35%)	1069 (27%)	1132 (28%)		13 (34%)	18 (44%)	8 (29%)
Obesity (≥30 kg/m^2^), *n* (%)	58 (33%)	1326 (34%)	1384 (34%)		12 (32%)	12 (29%)	8 (29%)
Body fat percentage,%, mean (sd) ^a^	33 (9)	32 (9)	32 (9)	0.45	35 (7)	33 (9)	31 (9)
Missing	79	733	812		21	16	10
Non-obese, *n* (%)	23 (23%)	886 (28%)	909 (28%)		3 (18%)	4 (16%)	7 (39%)
Pre-obese, *n* (%)	27 (27%)	722 (23%)	749 (23%)		6 (35%)	9 (36%)	4 (22%)
Obese, *n* (%)	49 (49%)	1553 (49%)	1602 (49%)		8 (47%)	12 (48%)	7 (39%)
6 min walk test, meters walked, mean (sd)	564 (100)	555 (104)	556 (104)	0.13	561 (87)	588 (101)	557 (86)
Missing	7	156	163		0	0	2
Grip strength, kg, mean (sd)	37.7 (12.4)	39.3 (13.3)	39.3 (13.3)	0.071	30.0 (5.8)	40.0 (14.8)	37.2 (13.3)
Missing	0	8	8		0	0	0
Knee extension, nm/kg, mean (sd)	133 (48)	142 (56)	141 (56)	0.069	116.8 (35.5)	139.8 (53.0)	133.5 (48.2)
Missing	17	443	460		2	1	3
Self-reported physical activity, meth/wk, median (iqr)	5.8 (0.5–18.4)	9.0 (0.0–24.0)	9.0 (0.0–24.0)	0.11	13.0 (1.3–21.8)	4.3 (0.4–12.0)	9.0 (3.0–26.3)
Missing	10	229	239		3	1	1
Smoking history, *n* (%)				0.046			
Never	107 (63%)	2677 (71%)	2784 (71%)		25 (69%)	30 (73%)	18 (67%)
Past	28 (16%)	436 (12%)	464 (12%)		7 (19%)	5 (12%)	3 (11%)
Current	35 (21%)	634 (17%)	669 (17%)		4 (11%)	6 (15%)	6 (22%)
Missing	8	147	155			0	1
Heavy/risky drinking ^b^, *n* (%)	65 (38%)	1370 (37%)	1435 (37%)	0.75	14 (40%)	13 (32%)	10 (37%)
Missing	9	215	224		3	1	1
Healthy lifestyle score ^c^, *n* (%)				0.086			
Unhealthy	98 (59%)	1843 (51%)	1941 (51%)		15 (43%)	22 (55%)	16 (59%)
Moderate	54 (32%)	1275 (35%)	1329 (35%)		18 (51%)	14 (35%)	6 (22%)
Healthy	15 (9.0%)	497 (14%)	512 (14%)		2 (5.7%)	4 (10%)	5 (19%)
Decade of primary cancer diagnosis, *n* (%)				<0.001			
1960–1970	9 (5.1%)	110 (2.8%)	119 (2.9%)		2 (5.3%)	0	2 (7.1%)
1971–1980	49 (28%)	576 (15%)	625 (15%)		8 (21%)	8 (20%)	10 (36%)
1981–1990	77 (43%)	1015 (26%)	1092 (27%)		19 (50%)	19 (46%)	11 (39%)
1991–2000	37 (21%)	1320 (34%)	1357 (33%)		9 (24%)	11 (27%)	4 (14%)
2001–	6 (3.4%)	873 (23%)	879 (22%)		0	3 (7.3%)	1 (3.6%)
Primary childhood cancer diagnosis, *n* (%)				<0.001			
Acute lymphoblastic leukemia	42 (24%)	1164 (30%)	1206 (30%)		11 (29%)	10 (24%)	5 (18%)
Acute myeloblastic leukemia/other leukemia	7 (3.9%)	153 (3.9%)	160 (3.9%)		1 (2.6%)	2 (4.9%)	2 (7.1%)
Non-hodgkin lymphoma	10 (5.6%)	268 (6.9%)	278 (6.8%)		2 (5.3%)	1 (2.4%)	3 (11%)
Hodgkin lymphoma	58 (33%)	416 (11%)	474 (12%)		19 (50%)	19 (46%)	6 (21%)
Central nervous system tumor	10 (5.6%)	543 (14%)	553 (14%)		1 (2.6%)	2 (4.9%)	1 (3.6%)
Neuroblastoma	11 (6.2%)	159 (4.1%)	170 (4.2%)		1 (2.6%)	1 (2.4%)	2 (7.1%)
Wilms tumor	6 (3.4%)	232 (6.0%)	238 (5.8%)		0	0	4 (14%)
Soft tissue sarcoma	3 (1.7%)	122 (3.1%)	125 (3.1%)		0	0	1 (3.6%)
Osteosarcoma	5 (2.8%)	161 (4.1%)	166 (4.1%)		0	0	2 (7.1%)
Ewing sarcoma family	8 (4.5%)	114 (2.9%)	122 (3.0%)		3 (7.9%)	1 (2.4%)	0
Retinoblastoma	4 (2.2%)	127 (3.3%)	131 (3.2%)		0	1 (2.4%)	0
Other	14 (7.9%)	435 (11%)	449 (11%)		0	4 (9.8%)	2 (7.1%)
Age at primary cancer diagnosis, years, mean (sd)	10.9 (6.1)	8.6 (5.6)	8.7 (5.7)	<0.001	13.3 (4.8)	12.1 (5.8)	8.5 (6.4)
Received radiation treatment, *n* (%)	139 (78%)	2078 (53%)	2217 (54%)	<0.001	30 (79%)	36 (88%)	18 (64%)
Received alkylating agents, *n* (%)	125 (70%)	2234 (57%)	2359 (58%)	0.002	26 (68%)	28 (68%)	20 (71%)
Received anthracyclines, *n* (%)	104 (58%)	2240 (58%)	2344 (58%)	0.92	22 (58%)	28 (68%)	17 (61%)
Received platinum, *n* (%)	21 (12%)	548 (14%)	569 (14%)	0.36	2 (5.3%)	2 (4.9%)	5 (18%)
Received epipodophyllotoxins, *n* (%)	58 (33%)	1405 (36%)	1463 (36%)	0.23	10 (26%)	19 (46%)	8 (29%)
Hematopoietic stem cell transplant, *n* (%)	17 (10%)	308 (8%)	325 (8%)	0.43	2 (5.3%)	6 (15%)	3 (11%)

^a^ Assessed with dual X-ray absorptiometry (DEXA). Categorized according to the obesity medicine association 2022 guidelines. For men, 25–29% was categorized as pre-obesity and ≥30% as obesity. For women, 30–34% was categorized as pre-obesity and ≥35% as obesity. ^b^ Risky alcohol consumption was defined as >3 drinks per day or >7 per week for women and >4 per day or >14 per week for men. ^c^ Derived from obesity, self-reported physical activity, risky drinking, and smoking [16]. ged = passed General Educational Development test. iqr = interquartile range. meth = metabolic equivalent of task hours. SMN = subsequent malignant neoplasm.

**Table 2:** Rate ratios for associations between lifestyle-associated variables and SMNs.

	Any SMN			Subsequent Breast Cancer	
	Univariate Analyses	Multivariable Model Incl. Treatment ^1^	Multivariable Model Incl. Diagnosis ^2^	Univariate Analyses	Treatment Model ^1^
	RR (95% CI)	RR (95% CI)	RR (95% CI)	RR (95% CI)	RR (95% CI)
Body mass index (bmi)					
Overweight (bmi ≥ 25–29.9)	1.5 (1.0–2.2)	1.3 (0.9–1.9)	1.3 (0.9–2.0)	1.5 (0.7–3.5)	NA
Obesity (bmi ≥ 30)	1.1 (0.7–1.6)	0.9 (0.6–1.4)	1.0 (0.7–1.5)	1.1 (0.5–2.6)	NA
Body fat percentage ^a^					
Pre-obesity vs. Normal	1.4 (0.8–2.4)	1.1 (0.6–1.9)	1.2 (0.7–2.1)	2.4 (0.6–9.6)	1.8 (0.4–7.2)
Obesity vs. Normal	1.2 (0.7–1.9)	0.7 (0.4–1.2)	0.9 (0.5–1.5)	1.6 (0.4–6.0)	0.7 (0.2–2.6)
Self-reported physical activity					
3–6 vs. 0–3 meth/wk	0.8 (0.5–1.3)	1.4 (0.8–2.3)	0.9 (0.6–1.4)	1.9 (0.8–4.3)	NA
≥6 vs. 0–3 meth/wk	0.7 (0.5–1.0)	1.1 (0.8–1.5)	0.8 (0.6–1.2)	1.1 (0.5–2.4)	NA
Cardiorespiratory fitness ^b^					
Quartile 4 vs. 1	1.1 (0.7–1.7)	1.4 (0.8–2.2)	1.0 (0.6–1.6)	0.8 (0.3–2.1)	NA
Quartile 3 vs. 1	1.3 (0.9–2.1)	1.7 (1.0–2.6)	1.3 (0.8–2.1)	1.4 (0.6–3.3)	NA
Quartile 2 vs. 1	1.0 (0.6–1.8)	1.2 (0.7–1.9)	1.0 (0.6–1.6)	0.9 (0.3–2.3)	NA
Grip strength					
High vs. Low	0.7 (0.5–1.1)	0.9 (0.5–1.6)	1.2 (0.7–2.1)	0.1 (0.04–0.3)	0.9 (0.4–1.8)
Moderate vs. Low	1.0 (0.7–1.5)	0.9 (0.6–1.3)	1.0 (0.7–1.5)	0.7 (0.4–1.5)	1.1 (0.3–4.4)
Knee extension					
High vs. Low	0.7 (0.5–1.1)	1.1 (0.7–1.8)	1.0 (0.6–1.6)	0.4 (0.2–1.03)	2.0 (0.8–5.4)
Moderate vs. Low	1.1 (0.7–1.6)	1.2 (0.8–1.8)	1.1 (0.7–1.7)	1.6 (0.8–3.6)	2.2 (1.0–5.0)
Never smoker	0.8 (0.6–1.1)	0.9 (0.7–1.3)	0.9 (0.6–1.3)	1.1 (0.5–2.2)	NA
Absence of risky drinking ^c^	1.0 (0.7–1.3)	0.7 (0.5–1.1)	0.9 (0.6–1.2)	0.9 (0.5–1.8)	NA
Healthy lifestyle score ^d^					
Moderate	0.9 (0.6–1.2)	0.9 (0.7–1.3)	0.9 (0.7–1.3)	1.9 (1.0–3.8)	NA
Healthy	0.6 (0.4–1.1)	0.9 (0.6–1.4)	0.8 (0.4–1.3)	0.5 (0.1–2.4)	NA

Multivariable models adjusted for sex, age at diagnosis and baseline assessment, treatment decade, and childhood cancer treatment ^1^ (anthracyclines, alkylating agents, platinum derivates, epipodophyllotoxins, and radiation exposure) or childhood cancer diagnosis ^2^. ^a^ Assessed with dual X-ray absorptiometry (DEXA). Categorized according to the obesity medicine association 2022 guidelines. For men, 25–29% was categorized as pre-obesity and ≥30% as obesity. For women, 30–34% was categorized as pre-obesity and ≥35% as obesity. ^b^ Assessed with the 6 min walk test. The 1st quartile is the least fit and the 4th quartile is the most fit group. ^c^ Risky alcohol consumption was defined as >3 drinks per day or >7 per week for women and >4 per day or >14 per week for men. ^d^ Derived from obesity, self-reported physical activity, risky drinking, and smoking [16]. bmi = body mass index. meth/wk = metabolic equivalent of task hours per week. NA = not applicable. RR = rate ratio. SMN = subsequent malignant neoplasm.

## Data Availability

The SJLIFE data that support the findings of this study are openly available at https://www.stjude.cloud/research-domains/cancer-survivorship. Data specific to this paper will be uploaded to https://zenodo.org concomitant with the publication of the manuscript.

## Supplementary Materials

The following supporting information can be downloaded at: https://www.mdpi.com/article/10.3390/cancers16050864/s1, Table S1. Distribution of types of first SMN in each individual during follow-up. Table S2. Mortality during follow-up in participants who did or did not develop an SMN during follow-up. Table S3. Cardiorespiratory fitness at baseline, as assessed by six-minute walk test, by survivors of Hodgkin lymphoma or other childhood cancer diagnosis.
