# Supplementary material for: Lifestyle and Subsequent Malignant Neoplasms in Childhood Cancer Survivors: A Report from the St. Jude Lifetime Cohort Study
Source: Cancers (Basel). 2024 Feb 21;16(5):864. doi: 10.3390/cancers16050864 (PMC10930858; doi:10.3390/cancers16050864)
Supplement: Supplementary file 1 [file cancers-16-00864-s001.zip › cancers-2851278-supplementary.pdf]

cancers
MDPI

Supplementary Table S1. Distribution of types of first SMN in each individual during follow-up.

SMN type	Frequency	Percent	Cumulative Frequency	Cumulative Percent
Breast	36	20.22	36	20.22
Gastrointestinal	26	14.61	62	34.83
Hematological	9	5.06	71	39.89
Meningioma (malignant)	3	1.69	74	41.57
Other central nervous system	7	3.93	81	45.51
Other Solid Tumors	27	15.17	108	60.67
Sarcoma	16	8.99	124	69.66
Thyroid	37	20.79	161	90.45
Urologic	17	9.55	178	100.00

Supplementary Table S2. Mortality during follow-up in participants who did or did not develop an SMN during follow-up.

Developed SMN	Died during follow-up, n (%)	Total
Yes	40 (22.5)	178
No	146 (37.5)	3894

Supplementary Table S3. Cardiorespiratory fitness at baseline, as assessed by six-minute walk test, by survivors of Hodgkin lymphoma or other childhood cancer diagnosis.

	1st quartile	2nd quartile	3rd quartile	4th quartile	Total
Hodgkin survivors	96 (20.9%)	104 (22.7%)	111 (24.2%)	148 (32.2%)	459
All other survivors	876 (25.4%)	905 (26.2%)	839 (24.3%)	830 (24.1%)	3,450

The 1st quartile is the least fit and the 4th quartile the most fit group.
